# Common gray and white matter abnormalities in schizophrenia and bipolar disorder

**DOI:** 10.1371/journal.pone.0232826

**Published:** 2020-05-07

**Authors:** Dong-Kyun Lee, Hyeongrae Lee, Kyeongwoo Park, Euwon Joh, Chul-Eung Kim, Seunghyong Ryu

**Affiliations:** 1 Department of Mental Health Research, National Center for Mental Health, Seoul, Republic of Korea; 2 Department of Clinical Psychology, National Center for Mental Health, Seoul, Republic of Korea; 3 Mental Health Research Institute, National Center for Mental Health, Seoul, Republic of Korea; 4 Department of Psychiatry, Chonnam National University Medical School, Gwangju, Republic of Korea; University of California, San Francisco, UNITED STATES

## Abstract

This study aimed to investigate abnormalities in the gray matter and white matter (GM and WM, respectively) that are shared between schizophrenia (SZ) and bipolar disorder (BD). We used 3T-magnetic resonance imaging to examine patients with SZ, BD, or healthy control (HC) subjects (aged 20–50 years, N = 65 in each group). We generated modulated GM maps through voxel-based morphometry (VBM) for T1-weighted images and skeletonized fractional anisotropy, mean diffusion, and radial diffusivity maps through tract-based special statistics (TBSS) methods for diffusion tensor imaging (DTI) data. These data were analyzed using a generalized linear model with pairwise comparisons between groups with a family-wise error corrected P < 0.017. The VBM analysis revealed widespread decreases in GM volume in SZ compared to HC, but patients with BD showed GM volume deficits limited to the right thalamus and left insular lobe. The TBSS analysis showed alterations of DTI parameters in widespread WM tracts both in SZ and BD patients compared to HC. The two disorders had WM alterations in the corpus callosum, superior longitudinal fasciculus, internal capsule, external capsule, posterior thalamic radiation, and fornix. However, we observed no differences in GM volume or WM integrity between SZ and BD. The study results suggest that GM volume deficits in the thalamus and insular lobe along with widespread disruptions of WM integrity might be the common neural mechanisms underlying the pathologies of SZ and BD.

## 1. Introduction

Accumulating evidence suggests that schizophrenia (SZ) and bipolar disorder (BD), classically distinct forms of psychosis, may share pathogenic mechanisms. Several studies have consistently reported a significant overlap in the clinical presentation [1,2], response to pharmacological treatment [3,4], and neurocognitive deficits between the two disorders [5,6]. Large-scale genetic studies have also provided evidence for common genetic causes between SZ and BD [7,8]. In light of this, exploring the common neuroanatomical characteristics of the two disorders could improve our understanding of the pathophysiological basis of the clinical and neurobiological continuum of psychosis.

Studies using magnetic resonance imaging (MRI) have provided evidence that there is an overlap in the abnormalities of brain structures in patients with SZ and BD. Several studies have reported gray matter (GM) volume deficits in both disorders. According to large-scale voxel-based morphometry (VBM) studies [9–11], patients with SZ show GM volume reduction in numerous cortical and subcortical regions, whereas patients with BD showed GM volume reduction which is restricted to the frontotemporal cortex. Meta-analyses of VBM studies have suggested that there is an overlap between SZ and BD in the areas affected by GM volume reduction including the frontal, temporal, cingulate and insular cortices, and the thalamus, with more extensive GM volume deficits in SZ than BD [12–14].

In addition to GM volume deficits, disruptions in the integrity of white matter (WM) have also been observed both in SZ and BD. A recent tract-based spatial statistics (TBSS) study observed that SZ and BD shared alterations of WM integrity in the fronto-temporal and callosal networks [15]. A recent meta-analysis of diffusion tensor imaging (DTI) studies also identified that the two disorders had common alterations in diffusion at the genu of the corpus callosum and in the fibers of the posterior cingulum [16]. However, several DTI studies directly comparing SZ and BD found no difference in WM integrity between the two disorders [15,17,18].

Taken together, structural neuroimaging studies have shown that widespread disruptions of WM integrity are consistent across SZ and BD, while GM volume deficits are more extensive in SZ than BD [19]. These findings suggest not only an anatomical overlap in the areas affected by structural abnormalities in SZ and BD, but also more extensive neuroanatomical pathology in SZ than in BD. However, only a minority of structural neuroimaging studies have directly compared SZ and BD, usually with small sample sizes [15,17,20,21]. There has also been no study comparing the two disorders in terms of both GM volume and WM integrity in the same sample. Therefore, an integrated analysis of the structural changes in GM and WM of patients with SZ and BD could provide more comprehensive insight into the psychosis continuum concept.

This study thus aimed to concurrently investigate GM and WM abnormalities in SZ and BD, and to explore neuroanatomical similarities and differences between the two disorders. To this end, we performed VBM and TBSS analyses of imaging data recorded in a single center from a relatively large number of SZ and BD, healthy control (HC) subjects compared to previous studies. To our knowledge, this is the first combined VBM and TBSS study which concurrently reports both alterations of GM volume and WM integrity in SZ and BD patients.

## 2. Materials and methods

### 2.1. Subjects

A psychologist (K.P.) interviewed all subjects using the Mini-International Neuropsychiatric Interview [22]. Patients who met the Diagnostic and Statistical Manual of Mental Disorders, Fourth Edition (DSM-IV) criteria for SZ and BD type I and II [23] were recruited from outpatient clinics of the National Center for Mental Health, Seoul, Republic of Korea. Inclusion criteria were as follows: (1) age 20–50 years; (2) duration of illness > 1 year; (3) clinically stable condition, defined by no exacerbation of psychotic symptoms and no change in general clinical state and medication for at least 3 months prior to the time of assessment. We excluded patients with a concurrent axis I diagnosis according to the DSM-IV, current or past neurological disease, any contraindication to MRI scan, or a physical condition that would render an MRI scan difficult to administer or interpret. The HCs consisted of volunteers from the local community who had the same age range and no history of psychiatric disorders. The same exclusion criteria for the patients was also applied to the HC group with regards to medical, neurological, and physical conditions. Data from 65 patients with SZ, 65 patients with BD, and 65 HCs were included in the final analysis. We assessed the overall level of the patients’ psychopathology and functioning using the 18-item Brief Psychiatric Rating Scale (BPRS-18) [24] and WHO Disability Assessment Schedule 2.0 (WHODAS 2.0) [25].

This study was initiated after approval of the Institutional Review Board of the National Center for Mental Health (IRB approval number: 116271-2017-26), and written informed consent was obtained from all subjects.

### 2.2. MRI data acquisition

The MRI data were acquired using a 3 Tesla MRI scanner (Ingenia CX; Philips, Erlangen) equipped with a 32-channel head coil at National Center for Mental Health. A high-resolution anatomical T1 weighted image was also acquired using a turbo field echo sequence [spin-echo repetition time (TR) = 9.8 ms, echo time (TE) = 4.6 ms, matrix size: 240 × 240, 170 sagittal slices, field of view (FOV) = 240 mm, slice thickness = 1 mm, flip angle = 8°]. The diffusion-weighted image was acquired using single-shot echo-planar images with the following parameters: acquisition matrix = 128 × 128, voxel size = 1.75 × 1.75 × 2 mm^3^, axial slices = 72, FOV = 224 × 224 mm^2^, TE = 60 ms, TR = 7696 ms, flip angle = 90°, slice gap = 0 mm, b value = 600 s/mm^2^. Diffusion-weighted images were acquired from 45 different directions. The baseline image without weighting was used [0, 0, 0].

### 2.3. T1 data processing

T1 images were processed using the VBM analysis pipeline with Statistical Parametric Mapping-version 8 (SPM8) software (Wellcome Department of Imaging Neuroscience, University College, London, UK). VBM is a fully automated image analysis technique which allows identification of tissue density in each voxel. The T1 images were segmented into GM, WM, and other tissues, including cerebrospinal fluid, using the Diffeomorphic Anatomical Registration Through Exponentiated Lie Algebra (DARTEL) algorithm in SPM8 software [26]. Modulation was then applied to the data to adjust for volume signal changes during normalization. Segmented T1 gray matter maps were warped to the standard space (1.5 × 1.5 × 1.5 mm Montreal Neurological Institute (MNI) 152 space) by multiplying the Jacobian determinants of the deformation fields that were obtained from the previously registered images using the “Normalize to MNI Space” option in SPM8 software. Spatial smoothing was applied using a Gaussian kernel of full width at half maximum (FWHM) 8 mm for the T1 tissue volume maps.

### 2.4. DTI data processing

DTI processing was performed using the FMRIB Software Library (FSL). First, motion artifacts and eddy current distortions through affine registration were corrected by taking the B0 volume as a reference using FSL’s Diffusion Toolbox. Then, the diffusion-weighted images were skull stripped using the “Brain Extraction Tool” within FSL. The fractional anisotropy (FA), axial diffusivity (AD), radial diffusivity (RD), and mean diffusivity (MD) images were obtained from the eigenvalues of the tensors using the DTIFIT program in FSL. Next, voxel-wise statistical analysis of the FA, AD, RD, and MD images was performed using the TBSS pipeline [27]. The FA images were aligned into the standard space (FMRIB58_FA, 1 × 1 × 1 mm MNI 152 space) using the nonlinear registration tool (FNIRT). Afterwards, a mean FA image was created and thresholded by an FA value of 0.2 to exclude peripheral tracts and GM regions. Each subject’s aligned FA images were then projected onto the skeleton by filling the skeleton with the highest FA values from the nearest relevant center of the fiber tracts. The same transformation and warped-field were applied to individual AD, RD, and MD images.

### 2.5. Statistical analyses

The VBM and TBSS analyses were performed using the SPM8 toolbox and FSL toolbox (randomize), respectively. Pairwise comparisons of the GM volumes and DTI parameters including FA, AD, RD, and MD were performed at the voxel-level for SZ vs. HC, BD vs. HC, and SZ vs. BD using a generalized linear model. We adjusted for age, sex, education, and intracranial volume as covariates to compare the GM volumes between SZ vs. HC and BD vs. HC. Age, sex, and education were adjusted to compare DTI parameters between SZ vs. HC and BD vs. HC. In addition, the GM volumes and DTI parameters controlling the duration of illness and therapeutic dose of antipsychotic drugs used were compared between SZ and BD in addition to the above-mentioned covariates. To adjust the voxel-wise multiple testings, we adopted the family-wise error (FWE) approach in the VBM and TBSS analyses. Significance thresholding for the TBSS analysis was determined using 10,000 permutations and threshold-free cluster-enhancement (TFCE) with the “2D” parameter settings [28]. Thereafter, to adjust the number of comparisons between pairs of groups (SZ vs. HC, BD vs. HC, and SZ vs. BD), additionally, we applied the Bonferroni correction to the FWE-corrected P value. Thus, FWE-corrected P values less than 0.017 were considered statistically significant. In addition, to present or interpret the findings in this manuscript, we set a cluster extent threshold of at least 80 mm^3^ contiguous volumes because of surplus brain regions.

## 3. Results

### 3.1. Demographic and clinical characteristics

Table 1 summarizes the demographic and clinical characteristics of the SZ, BD, and HC groups. There were no significant differences in age or sex among the 3 groups, but HC had a significantly higher level of education than SZ (P < 0.001) and BD (P < 0.001). Treatment dose of antipsychotic drugs was significantly higher in SZ than in BD (t = 6.26, P < 0.001). The SZ group showed significantly higher scores in the BPRS-18 (t = 4.17, P < 0.001) and WHODAS 2.0 (t = 2.80, P = 0.006) than the BD group. With regards to the subtype of BD, there were 54 patients with BD type I and 11 patients with BD type II. Forty-one (63.08%) of patients with BD had a history of psychosis.

**Table 1:** Demographic and clinical characteristics.

Variables^a^	Healthy control (N = 65)	Schizophrenia (N = 65)	Bipolar disorder (N = 65)	Statistics^b^
Age, y	34.52 ± 8.93	36.98 ± 7.88	35.06 ± 9.24	F = 1.44, P = 0.240
Sex (male / female), n	28 / 37	36 / 29	29 / 36	χ^2^ = 2.34, P = 0.310
Education, y	14.78 ± 2.55	13.09 ± 2.10	13.78 ± 1.94	F = 9.60, P < 0.001
Handedness (right / left), n	57 / 8	62 / 3	58 / 7	χ^2^ = 2.57, P = 0.277
Duration of illness, y	-	15.52 ± 7.52	13.45 ± 8.41	t = 1.48, P = 0.140
Medication				
Antipsychotics, n	-	65	50	χ^2^ = 16.96, P < 0.001
chlorpromazine equivalent dose, mg	-	757.21 ± 433.91	331.90 ± 334.12	t = 6.26, P < 0.001
Mood stabilizers, n	-	18	50	χ^2^ = 31.58, P < 0.001
valproate / lithium / lamotrigine, n	-	13 / 5 / 1	30 / 23 / 6	-
Antidepressants, n	-	19	12	χ^2^ = 2.08, P = 0.149
BPRS-18 total score	-	33.17 ± 6.09	28.02 ± 7.93	t = 4.17, P < 0.001
WHODAS 2.0 total score	-	15.40 ± 6.73	11.96 ± 7.28	t = 2.80, P = 0.006

^a^ Data are shown as mean ± SD or N.

^b^ ANOVA, chi-square test, or independent t test.

Abbreviations: BPRS-18, 18-item Brief Psychiatric Rating Scale; WHODAS 2.0, WHO Disability Assessment Schedule 2.0.

### 3.2. Comparisons of GM volume between groups

**Fig 1:**
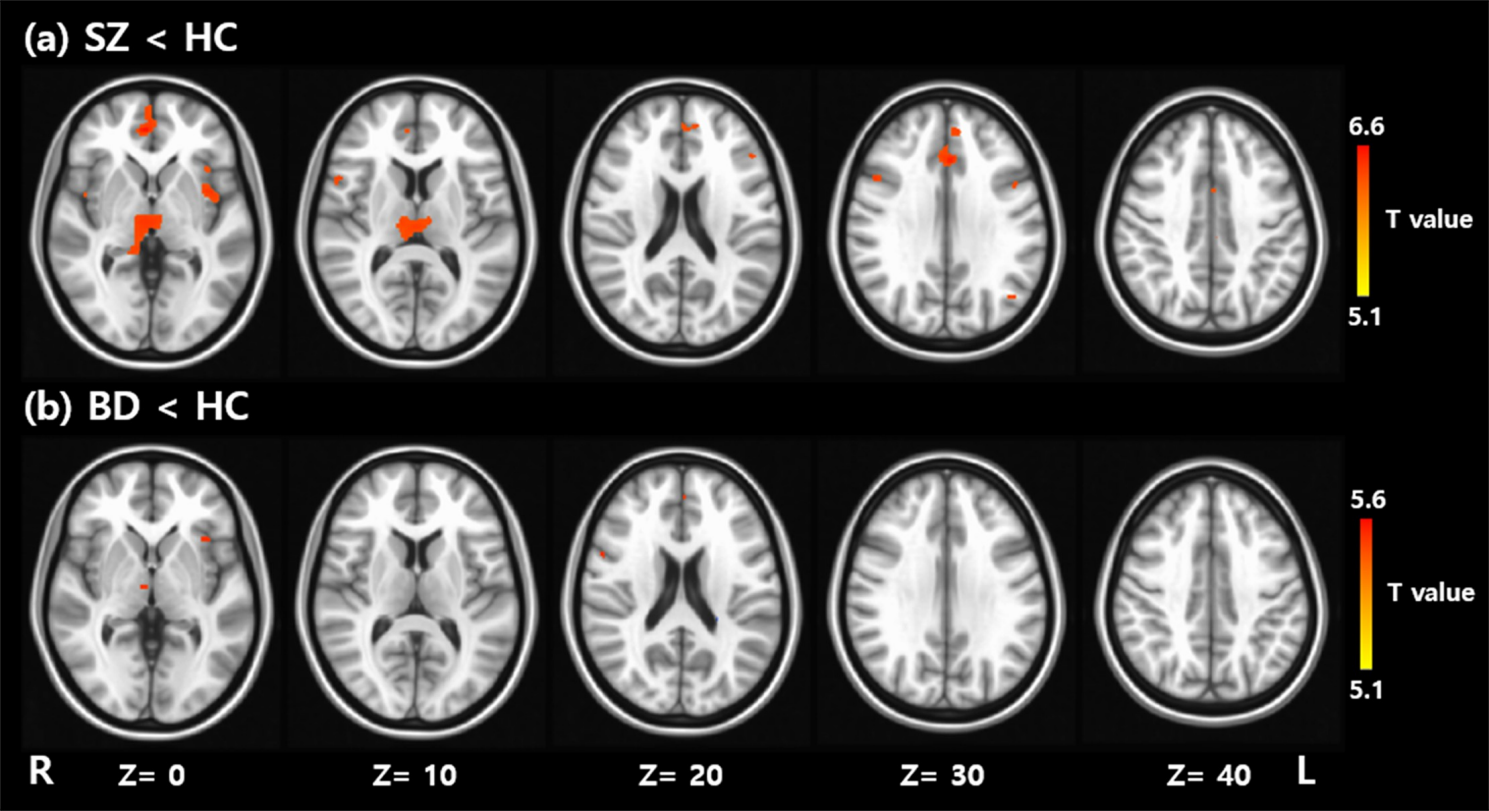
Voxel based morphometry analyses identified regions which show significant differences in gray matter volume between schizophrenia (SZ) and Healthy Control (HC) (a), and between Bipolar Disorder (BD) and HC (b). Family-wise error corrected P < 0.017.

GM volume deficits were explored between the SZ and BD groups (Fig 1). Compared to HC, SZ showed GM volume reductions in multiple cortical and subcortical areas, including the frontal (bilateral middle orbital gyrus, bilateral inferior frontal gyrus, left anterior cingulate cortex, and left medial frontal gyrus), temporal (bilateral insular lobe and bilateral olfactory cortex, and right hippocampus), and parietal (left angular gyrus) areas, as well as in the thalamus and caudate nucleus (Table 2). Peak voxel was observed in the thalamus. However, BD showed GM volume deficits only in the right thalamus and left insular lobe (Table 2), and moreover, their cluster sizes were relatively small. The right thalamus had the voxel with the highest significance. Taken together, the right thalamus and left insular lobe were the common areas that showed GM volume deficits in both SZ and BD groups. In addition, a direct comparison between SZ and BD revealed no significant difference in GM volume. All significant findings in the VBM analyses, regardless of the voxel cluster threshold, are presented in S1 Table.

**Table 2:** Pairwise comparisons of gray matter volume between SZ, BD, and HC groups^a^.

Anatomical region	Side	T max	Peak coordinates (MNI)			Cluster size (mm^3^)
			x	y	z	
SZ vs. HC						
Thalamus	L/R	5.812	0	-19	7	5268
Middle orbital gyrus	R	6.476	4	47	-2	2464
Inferior frontal gyrus	L	6.597	-39	17	-14	2440
Anterior cingulate Cortex	L	6.283	-39	17	-14	1252
Insular lobe	L	6.007	-38	5	3	864
Medial frontal gyrus	L	5.641	-2	48	22	611
Caudate nucleus	L	5.839	-6	18	-6	513
Olfactory cortex	R	5.682	27	8	-14	398
Insular lobe	R	5.862	38	18	-15	375
Angular gyrus	L	5.351	-45	-67	28	314
Inferior frontal gyrus	R	5.621	54	14	7	226
Caudate nucleus	R	5.352	9	20	-5	169
Hippocampus	R	5.614	30	-9	-17	101
Middle orbital gyrus	L	5.360	2	59	-14	101
Inferior frontal gyrus	L	5.494	-46	9	28	94
Inferior frontal gyrus	R	5.511	50	14	30	88
Inferior frontal gyrus	L	5.634	-48	29	19	84
BD vs. HC						
Thalamus	R	5.285	8	-12	1	155
Insular lobe	L	5.529	-38	14	-15	84

^a^ Family-wise error corrected P < 0.017

That all MNI coordinates of maximum t values are selected in the significant region.

That there was no significant difference in gray matter volume between the SZ and BD groups.

Abbreviations: SZ, Schizophrenia. BD, Bipolar Disorder. HC, Healthy Control. MNI, Montreal Neurological Institute. L, Left. R, Right.

### 3.3. Comparisons of WM integrity between groups

**Fig 2:**
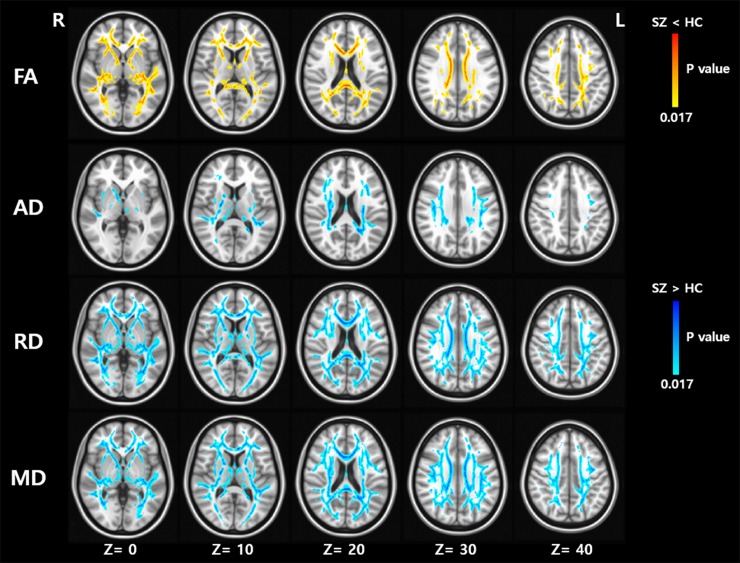
Tract-based special statistics analyses found widespread areas showing diffusion abnormalities (Fractional Anisotropy, FA; Axial Diffusivity, AD; Radial Diffusivity, RD; Mean Diffusivity, MD) in patients with schizophrenia (SZ) compared to healthy controls (HCs). Family-wise error corrected P < 0.017.

**Fig 3:**
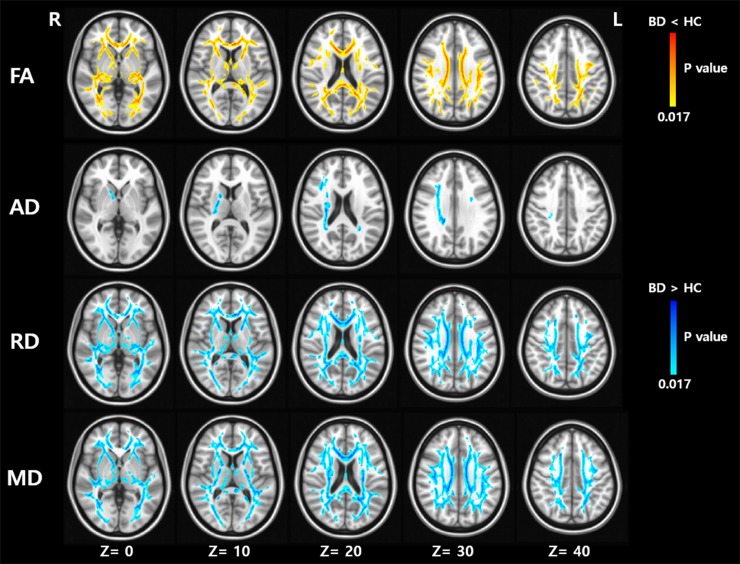
Tract-based special statistics analyses found widespread areas showing diffusion abnormalities (Fractional Anisotropy, FA; Axial Diffusivity, AD; Radial Diffusivity, RD; Mean Diffusivity, MD) in patients with bipolar disorder (BD) compared to healthy controls (HCs). Family-wise error corrected P < 0.017.

Disruptions of WM integrity were explored in the SZ and BD groups (Figs 2 and 3). Widespread alterations in diffusion were identified in both the SZ and BD groups (Tables 3 and 4). In terms of FA, the affected voxels occupied 31.0% of the areas within the WM skeleton in SZ and 36.2% of the areas in BD. In comparison with HC, both disorders showed a significant decrease in FA and an increase in AD, RD, and MD in several major WM tracts. In terms of common areas showing WM alterations in both SZ and BD, FA decreased in the body and splenium of the corpus callosum, the body of the fornix, the left posterior thalamic radiation, right anterior limb and retrolenticular part of the internal capsule, and the right external capsule. While both SZ and BD showed an increase in AD only in the right posterior limb of the internal capsule, RD increased in both disorders in the body of the corpus callosum, the bilateral retrolenticular parts of the internal capsule, the left posterior corona radiata, and the right external capsule. In addition, both SZ and BD showed an increase in MD in the bilateral superior longitudinal fasciculi, left superior corona radiata, right posterior corona radiata, right anterior corona radiata, and right external capsule. Direct comparisons of DTI parameters between SZ and BD revealed no significant differences. All significant findings in the TBSS analyses, regardless of the voxel cluster threshold, are presented in S2–S5 Tables.

**Table 3:** Comparisons of diffusion indexes between SZ and HC groups^a^.

Anatomical region	Side	T max	Peak coordinates (MNI)			Cluster size (mm^3^)
			x	y	z	
Fractional Anisotropy (FA) SZ vs. HC						
Anterior corona radiata	L	5.424	-18	34	16	2268
Posterior thalamic radiation	L	4.878	-26	-62	15	481
Fornix (cres)	L	4.806	-28	-25	-8	370
Anterior limb of internal capsule	R	4.557	21	19	7	353
Anterior corona radiata	R	3.975	13	32	-8	339
Body of corpus callosum		5.247	7	11	24	336
Fornix (column and body)		4.666	0	-1	15	293
Retrolenticular part of internal capsule	L	4.983	-29	-22	0	287
Body of corpus callosum		5.212	9	-28	25	255
External capsule	R	4.054	28	19	2	130
Splenium of corpus callosum		3.853	-5	-37	20	121
Sagittal stratum	L	4.060	-39	-44	-7	118
Splenium of corpus callosum		4.543	26	-53	15	109
Anterior limb of internal capsule	L	4.304	-21	10	13	100
Retrolenticular part of internal capsule	R	4.815	34	-30	5	96
Anterior limb of internal capsule	L	3.557	-20	18	4	83
Axial Diffusivity (AD) SZ vs. HC						
Posterior corona radiata	R	4.4447	28	-31	19	541
Superior longitudinal fasciculus	R	3.4658	37	-16	27	127
Posterior limb of internal capsule	R	3.5328	20	-6	9	84
Superior longitudinal fasciculus	L	4.3845	-41	-9	26	80
Radial Diffusivity (RD) SZ vs. HC						
Superior corona radiata	L	6.0182	-17	-9	39	3218
Body of corpus callosum		5.3067	10	13	24	1219
Retrolenticular part of internal capsule	L	4.9983	-30	-24	-1	602
Retrolenticular part of internal capsule	R	5.2397	37	-33	5	463
Fornix (cres)	L	4.6318	-27	-26	-8	183
Posterior corona radiata	L	4.6159	-27	-36	25	142
Splenium of corpus callosum		4.6734	26	-53	15	141
Anterior limb of internal capsule	L	3.9972	-20	18	4	93
External capsule	R	3.9933	29	15	3	90
Posterior thalamic radiation	R	3.9213	35	-62	1	87
Mean Diffusivity (MD) SZ vs. HC						
Superior corona radiata	L	5.7383	-18	16	36	3726
Posterior corona radiata	L	5.036	-26	-34	27	1078
Anterior corona radiata	L	4.9447	-17	39	-1	752
Posterior corona radiata	R	4.2994	28	-41	25	314
Anterior corona radiata	R	4.8575	25	23	7	305
Superior longitudinal fasciculus	R	4.0915	38	-14	29	293
Sagittal stratum	L	4.8634	-40	-42	-7	141
Retrolenticular part of internal capsule	L	3.8339	-33	-33	7	132
Superior longitudinal fasciculus	L	5.5257	-40	-42	13	112
External capsule	R	5.6883	32	11	-1	103
Posterior limb of internal capsule	R	3.8928	25	-7	18	92

^a^ Family-wise error corrected P < 0.017

That all MNI coordinates of maximum t values are selected in the significant region.

Abbreviations: SZ, Schizophrenia. HC, Healthy Control. MNI, Montreal Neurological Institute. L, Left. R, Right.

**Table 4:** Comparisons of diffusion indexes between BD and HC groups^a^.

Anatomical region	Side	T max	Peak coordinates (MNI)			Cluster size (mm^3^)
			x	y	z	
Fractional Anisotropy (FA) BD vs. HC						
Body of corpus callosum		6.409	-9	17	22	5205
Posterior thalamic radiation	L	5.472	-26	-67	15	605
Anterior limb of internal capsule	R	4.911	21	19	5	488
External capsule	R	4.918	29	15	3	412
Splenium of corpus callosum		4.084	25	-52	22	338
External capsule	L	3.701	-30	10	-8	296
Cingulum	L	4.127	-10	-20	34	155
Posterior corona radiata	L	4.393	-26	-29	26	138
Fornix (cres)	L	4.551	-26	-32	-3	97
Retrolenticular part of internal capsule	R	3.906	30	-20	-1	97
Fornix (cres)	R	4.822	28	-27	-6	92
Axial Diffusivity (AD) BD vs. HC						
Superior corona radiata	R	4.2171	24	-8	37	381
Posterior limb of internal capsule	R	3.6601	26	-20	12	134
Superior corona radiata	L	5.0367	-27	1	31	106
Posterior limb of internal capsule	R	4.465	26	-8	18	83
Radial Diffusivity (RD) BD vs. HC						
Body of corpus callosum		5.992	-10	17	23	8506
External capsule	R	5.048	29	16	3	1163
Posterior thalamic radiation	L	5.752	-26	-67	15	533
Retrolenticular part of internal capsule	R	4.572	35	-29	5	442
Posterior corona radiata	L	4.697	-26	-32	25	356
Cingulum	L	5.137	-9	-35	33	223
Superior corona radiata	R	4.51	27	-14	33	183
Cingulum	R	4.478	12	-50	18	118
Retrolenticular part of internal capsule	L	3.43	-32	-28	4	97
Mean Diffusivity (MD) BD vs. HC						
Superior corona radiata	L	5.744	-18	-3	41	5391
Superior corona radiata	R	5.77	28	7	28	1844
Superior longitudinal fasciculus	L	5.232	-35	-41	31	971
Anterior corona radiata	R	4.659	18	37	7	737
Posterior thalamic radiation	R	4.17	29	-57	17	635
Posterior corona radiata	R	4.744	29	-44	26	520
Body of corpus callosum		4.648	12	-28	27	189
Anterior limb of internal capsule	R	3.808	21	19	6	137
External capsule	R	5.081	31	12	-2	129
Superior longitudinal fasciculus	R	4.949	33	2	20	99
External capsule	L	4.173	-24	21	3	91

^a^ Family-wise error corrected P < 0.017

That all MNI coordinates of maximum t values are selected in the significant region.

Abbreviations: BD, Bipolar Disorder. HC, Healthy Control. MNI, Montreal Neurological Institute. L, Left. R, Right.

## 4. Discussion

In this study, we observed more extensive GM volume deficits in SZ than those in BD, in addition to significant alterations in WM integrity which showed substantial overlap across both SZ and BD. In the VBM analysis, compared to HC, SZ exhibited GM volume deficits in multiple cortical and subcortical areas. In contrast, BD showed GM volume deficits only in limited regions, including the right thalamus and left insular lobe, both of which were common areas in both SZ and BD for GM volume deficit. On the other hand, TBSS analyses revealed alterations in diffusion in both SZ and BD in about one third of the areas of a WM skeleton which represents all major WM tracts, with widespread overlap in the affected areas across the two disorders. Meanwhile, direct comparisons between SZ and BD did not reveal any significant differences in GM volume or WM integrity in neither the VBM, nor the TBSS analysis.

### 4.1. GM alterations occur in common areas in SZ and BD but are more widespread in SZ

In this study, compared to HC, in SZ, the GM volume deficits were widely distributed across the thalamus, middle orbital gyrus, inferior frontal gyrus, anterior cingulate cortex, insular lobe, medial frontal gyrus, caudate nucleus, olfactory cortex, angular gyrus, and hippocampus. These affected regions were mostly consistent with those reported by previously published large-sized VBM analyses and meta-analyses which have reported that patients with SZ show GM volume deficits throughout the frontal, temporal and parietal lobes; cingulate and insular cortices; and thalamus [9,10,29–31]. However, reports of volume change of caudate nucleus in SZ has been controversial [12,32,33]. Evidence suggests that long-term antipsychotic treatment might have a significant effect on the caudate nucleus volume of patients with SZ [34–36]. In contrast, in patients with BD as compared with HC, we observed only local reductions in GM volume, which were limited to the right thalamus and left insular lobe. As above-mentioned, GM volume reduction in the thalamus has mainly been observed in patients with SZ, but findings in patients with BD have been inconsistent [20,37–39]. However, a large-sized meta-analysis reported a significant decrease in thalamus volume in patients with BD compared with HC [40]. In addition, several meta-analyses have reported the association between GM volume deficits in insular lobe and BD [12,14,41], which is in agreement with our findings.

Overall, our VBM analyses could draw the conclusion that SZ and BD share common GM volume deficit in the right thalamus and left insular lobe, which is consistent with the results of previous meta-analyses. A previously published meta-analysis reported GM volume deficits in the thalamus is common in both SZ and BD [42]. With regard to the insular lobe, GM volume in the insular lobes has been consistently reported to decrease in both SZ and BD [12,42]. Moreover, a VBM meta-analysis across 6 diverse diagnostic groups including SZ, BD, depression, addiction, obsessive-compulsive disorder, and anxiety also identified the insular lobes to be major regions showing common GM loss across diagnoses [43]. Moreover, in this study, a direct comparison between SZ and BD showed no significant difference in GM volume. A small number of studies have directly compared GM volume between SZ and BD, but distinct structural features of the two disorders have not been conclusively identified. Some studies found no difference in GM volume between SZ and BD [21,44,45], whilst other studies have reported that compared to BD, SZ patients showed smaller GM volume in some subcortical regions including the thalamus, putamen, hippocampus, and amygdala [9,37,46] or even in widespread cortical regions [20].

Recent series of meta-analyses from the ENIGMA consortium have identified several cortical and subcortical regions associated with GM volume reduction in patients with SZ and BD [40,47–49]. Putting the findings of these studies together, it can be inferred that SZ is associated with more extensive deficits compared to BD, and that there is an overlap in the affected regions between the two disorders. Our VBM analyses performed simultaneous comparisons between SZ vs. HC and between BD vs. HC and confirmed that GM volume reductions are typically more extensive in SZ than in BD. In addition, patterns of the overlap between patients with SZ and those with BD suggest that GM volume deficits in the thalamus and insular lobe might be common neurobiological substrates across SZ and BD. Considering that these areas have been structurally and functionally linked to delusions and hallucinations [50–52], these volumetric alterations might be particularly relevant to the pathophysiology of psychotic BD, which warrants the need for further analyses to explore the neural substrates that are common to both SZ and psychotic BD, or those that differ between psychotic and non-psychotic BD. Finally, the finding that compared with HC, patients with SZ showed a widespread reduction in GM volume, whereas those with BD showed only local deficits mostly limited to the thalamus and insular lobe, possibly indicates that in considerable brain areas, relative to BD, SZ might be associated with more severe GM volume deficits. However, unexpectedly, we observed no statistically significant difference in GM volumes between the two disorders, implying that BD might be associated with GM volume reduction, and that the magnitude of this reduction might be between that observed in SZ and HC. Therefore, further studies with sufficient statistical power to detect a difference between SZ and BD are warranted in future.

### 4.2. Widespread compromise of WM integrity occurs in both SZ and BD

In this study, we concurrently investigated multiple DTI parameters including FA, AD, RD, and MD, which allowed us to comprehensively understand microstructural alterations in the WM of SZ and BD patients. FA measures how much one direction prevails over others within a particular voxel [53]. As a marker of axonal integrity, FA is highly sensitive for detecting microstructural changes in the WM. AD and RD measure the amount of diffusion that occurs along the principal axis of the diffusion tensor and perpendicularly to it [54]. AD increases with brain maturation after birth, whereas it decreases in the case of axonal injury. RD is a putative marker of myelination, whereas an increased RD is suggestive of de- or dys- myelination. MD is an inverse measure of membrane density which represents the magnitude of diffusion independent of direction, while retaining sensitivity to cellularity, edema, and necrosis [55]. A combined interpretation of these DTI parameters may allow us to better characterize the WM microstructure in SZ and BD. Our TBSS analyses identified several major WM tracts which showed decreased FA or increased AD, RD, or MD in SZ and BD compared to HCs. In particular, in some WM areas, a decrease in FA was coupled with an increase in RD or MD, which could be interpreted as a consequence of disrupted brain connections or demyelination. The directions of alteration in these DTI parameters were largely consistent with findings of previous DTI studies into SZ and BD [15,56,57]. In addition, direct comparisons between SZ and BD revealed no significant difference in any DTI parameters between the two disorders.

We observed that compared to HCs, significant decreases in FA were widespread over around 30% of areas of the whole-brain WM skeleton in both SZ and BD. We also detected a widespread increase in RD and MD, as well as a local increased in AD. Regarding SZ, decreases in FA were coupled with increases in RD in areas of the corpus callosum, fornix, and internal and external capsules, which indicates probable damage to myelination and barriers perpendicular to the main axis of the axons. In addition, decreases in FA were coupled with increases in MD in the areas of the internal and external capsules, which is suggestive of damage to the boundaries which are required for diffusion. Our findings related to SZ are largely in agreement with the results of a recent large-scale meta-analysis of DTI data which showed significant global decreases in FA across the WM skeleton, with the largest effects size in the anterior corona radiata and corpus callosum, as well as widespread increases in RD and MD [58]. With regards to BD, decreases in FA were coupled with increases in RD in the body of the corpus callosum, the posterior thalamic radiation, posterior corona radiata, cingulum, and areas of the internal and external capsules. Decreases in FA were also coupled with increases in MD in the body of the corpus callosum and the external capsule. Although there has not always been agreement in terms of the findings of previous DTI studies into BD [16,59], relevant microstructural changes have been suggested in various WM regions including the corpus callosum, cingulum, longitudinal fasciculus, uncinate fasciculus, thalamic radiation, and the corona radiata. A recent TBSS study reported decreases in FA in widespread WM regions including the corpus callosum, corona radiata, internal capsule, and the longitudinal fasciculus, as well as increases in RD and MD, which is consistent with our findings with respect to BD [15].

Our TBSS analysis revealed that compared to HC, SZ and BD shared microstructural abnormalities in multiple WM regions including the corpus callosum, corona radiata, superior longitudinal fasciculus, posterior thalamic radiation, fornix, and the internal and external capsules. In particular, alterations in diffusion were consistently located in the corpus callosum for both diseases, suggesting that aberrant interhemispheric communication might represent a common neural underpinning of the two psychotic disorders. The corpus callosum contains axon fibers connecting the bilateral frontal cortices, WM integrity of which is related to cognitive performance in various domains including sustained attention, processing speed, and problem solving abilities, which are frequently impaired in both SZ and BD [60,61]. DTI studies have provided evidence for WM abnormalities in the corpus callosum in the two disorders [17]. In addition to alterations in the corpus callosum, our findings indicate impaired integrity of the WM microstructure between the fronto-temporal and fronto-subcortical regions in both conditions, which are areas linked to emotional and cognitive processes [62,63]. A recent TBSS study reported that compared to HCs, patients with both SZ and BD showed a widespread reduction in FA and a significant increase in RD, AD, and MD in major WM tracts, especially fronto-temporal and callosal networks [15], which is mostly in agreement with our findings. A voxel-based meta-analysis of DTI studies also showed that both types of patients exhibited significantly reduced FA in the genu of the corpus callosum and the left posterior cingulum fibers compared to HCs [16]. This TBSS study and the meta-analysis also reported no difference in diffusion between SZ and BD. Taken together, findings of our TBSS analysis indicate widespread impairment of WM microstructural integrity in both SZ and BD, suggesting that extensive structural disconnect between brain networks might be a common neural underpinning of psychotic disorders.

### 4.3. Limitations

This study has some methodological limitations. The study subjects were heterogeneous in terms of demographic and clinical characteristics. First, patients with SZ and BD showed a wide range in the duration of illness and had received various types and doses of antipsychotics, mood stabilizers, or antidepressants. Considering evidence of studies on the effects of aging and medication on brain structure [31,64], we could not exclude the possibility that the heterogeneity of subjects might have influenced the results. Second, the BD group included patients with all types of BD regardless of whether they were type I, type II, or had a history of psychosis. Some neuroimaging studies have reported characteristic GM and WM alterations according to BD subtypes [65,66]. Notwithstanding, this study did not cover the effect of BD subtypes on structural abnormalities because subgrouping patients with BD by BD subtypes can lead to the sample size difference in the SZ and HC groups, which might complicate the interpretation of group comparisons. Instead, we are planning additional studies to compare SZ and BD subtypes with regard to GM volume and WM integrity. Third, patients with SZ or BD had significantly lower levels of education than HCs, though we controlled for this covariate statistically in comparisons with HCs. Finally, the results of VBM and TBSS analyses could not be interpreted in an integrated manner for the affected brain regions. In this regard, a voxel-based assessment of WM volume might help to integrate the neuroanatomical comparisons. Considering these limitations, careful interpretation of our findings is necessary.

### 4.4. Conclusion

In conclusion, compared with HC, SZ had extensive deficits in GM volume than BD. However, SZ and BD shared widespread disruptions in WM integrity, as well as regional decreases of GM volume in the thalamus and insular lobe. These findings suggest that the two disorders might both involve widespread impairment of WM microstructural integrity, but with more extensive GM damage in SZ pathogenesis than BD. Further studies are warranted into both SZ and BD as brain network disorders.

## Supporting information

S1 Table Brain areas showing a significant difference in gray matter volume among schizophrenia, bipolar disorder and healthy control groups at the level of FWE-corrected P < 0.017. (XLSX)

S2 Table Brain areas showing a significant difference in fractional anisotropy among schizophrenia, bipolar disorder and healthy control groups at the level of FWE-corrected P < 0.017. (XLSX)

S3 Table Brain areas showing a significant difference in axial diffusivity among schizophrenia, bipolar disorder and healthy control groups at the level of FWE-corrected P < 0.017. (XLSX)

S4 Table Brain areas showing a significant difference in radial diffusivity among schizophrenia, bipolar disorder and healthy control groups at the level of FWE-corrected P < 0.017. (XLSX)

S5 Table Brain areas showing a significant difference in mean diffusivity among schizophrenia, bipolar disorder and healthy control groups at the level of FWE-corrected P < 0.017. (XLSX)
